# Invasive *Trichosporon* Infection in Solid Organ Transplant Recipients: A Single-Center Experience in Madrid, Spain

**DOI:** 10.3390/jof11120862

**Published:** 2025-12-05

**Authors:** Antonio Ramos-Martínez, Oscar Zaragoza, Andrea Gutiérrez-Villanueva, Lina Parra-Ramirez, Mireia Cantero-Caballero, Reyes Iranzo-Valero, Isabel Sánchez-Romero, Itziar Diego-Yagüe, Rosalía Laporta-Hernández, Ana Fernández-Cruz, Manuel Gómez-Bueno, Elena Múñez-Rubio, Nuria Novoa-Valentín, Carlos Martín-Martín, Ana Duca, José Luis Lucena-de la Poza, Jorge Calderón-Parra

**Affiliations:** 1 Facultad de Medicina, Universidad Autónoma de Madrid (UAM), 28029 Madrid, Spain; aramos220@gmail.com; 2 Laboratorio de Referencia e Investigación en Micología, Centro Nacional de Microbiología, Instituto de Salud Carlos III, Majadahonda, 28222 Madrid, Spain; ozaragoza@isciii.es; 3 Center for Biomedical Research in Network in Infectious Diseases CIBERINFEC (CB21/13/00105), Instituto de Salud Carlos III, 28029 Madrid, Spain; 4 Unidad de Enfermedades Infecciosas, Servicio de Medicina Interna, Hospital Universitario Puerta de Hierro, 28222 Majadahonda, Spain; itziardiego@gmail.com (I.D.-Y.); anafcruz999@gmail.com (A.F.-C.); elmuru@gmail.com (E.M.-R.); 5 Servicio de Medicina Preventiva, Hospital Universitario Puerta de Hierro, 28222 Majadahonda, Spain; linamarcela.parra@salud.madrid.org (L.P.-R.); mireia.cantero@salud.madrid.org (M.C.-C.); 6 Servicio de Anestesiología, Hospital Universitario Puerta de Hierro, 28222 Majadahonda, Spain; mariareyes.iranzo@salud.madrid.org; 7 Servicio de Microbiología, Hospital Universitario Puerta de Hierro, 28222 Majadahonda, Spain; 8 Servicio de Neumología, Hospital Universitario Puerta de Hierro, 28222 Majadahonda, Spain; rosalia.laporta@salud.madrid.org; 9 Servicio de Cardiología, Hospital Universitario Puerta de Hierro, 28222 Majadahonda, Spain; manuelfrancisco.gomez@salud.madrid.org; 10 Servicio de Cirugía de Tórax, Hospital Universitario Puerta de Hierro, 28222 Majadahonda, Spain; nuriamaria.novoa@salud.madrid.org; 11 Servicio de Cirugía Cardiaca, Hospital Universitario Puerta de Hierro, 28222 Majadahonda, Spain; carlos.martin.martin@madrid.org; 12 Unidad de Trasplante Hepático, Servicio de Medicina Interna, Hospital Universitario Puerta de Hierro, 28222 Majadahonda, Spain; ana.duca@salud.madrid.org; 13 Servicio de Cirugía General y Digestiva, Hospital Universitario Puerta de Hierro, 28222 Majadahonda, Spain; joseluis.lucena@salud.madrid.org; 14 Instituto de Investigación Sanitaria Puerta de Hierro-Segovia de Arana (IDIPHISA), Joaquín Rodrigo 2, 28222 Majadahonda, Spain; jorge050390@gmail.com

**Keywords:** *Trichosporon*, organ transplant, invasive fungal infection, fungemia, voriconazole

## Abstract

Background: Invasive fungal infections are a major threat in solid organ transplant (SOT) recipients. *Trichosporon* spp. are emerging yeasts associated with high mortality and therapeutic difficulties. Methods: Retrospective study of SOT recipients with invasive *Trichosporon* spp. infection at a tertiary hospital in Spain (2017–2025) was performed. Demographic, clinical, microbiological, and outcome data were analyzed. Results: Sixteen patients (56.2% male; median age 54 years) (lung: eight; heart: five; liver: three) with infection due to *Trichosporon austroamericanum* were included. Hospital mortality was 50% (8 out of 16 patients). The infection originated at the surgical site in 14 cases (87.5%), with progression to fungemia in 6 patients, all of whom died. Univariate analysis identified breakthrough infection (*p* = 0.010), concomitant antibiotics (*p* = 0.026), high-dose corticosteroid therapy (*p* = 0.020), and ICU admission at diagnosis (*p* = 0.001) as risk factors for mortality. All strains exhibited favorable in vitro susceptibility to voriconazole, isavuconazole, posaconazole and amphotericin B and high MICs for echinocandins. Conclusions: Invasive *Trichosporon* spp. infection in SOT recipients is linked to considerable mortality, especially in surgical site infections complicated by fungemia. Mortality is associated with severe immunosuppression, breakthrough infection, concomitant antibiotics, ICU admission, and delayed diagnosis. The combined administration of broad-spectrum antibiotics and echinocandins was associated with mortality.

## 1. Introduction

Invasive fungal infections (IFIs) are a significant cause of morbidity and mortality in solid organ transplant (SOT) recipients [1]. While *Candida* spp. and *Aspergillus* spp. remain the most common pathogens in this setting, several factors have led to infections with more diverse pathogens [2]. Among these, *Trichosporon* spp. are ubiquitous basidiomycete yeasts that colonize the gastrointestinal tract, respiratory tract, and skin. This emerging infection primarily affects patients with hematological malignancies, solid organ transplants, prolonged neutropenia, or invasive devices such as central venous catheters who have received broad-spectrum antibiotic treatment [3,4,5,6,7]. *Trichosporon* spp. infections in solid organ transplant recipients are very rare in Spain. In fact, only one series of six cases in lung transplant recipients and one case of surgical wound infection have been reported [4,8]. Globally, the incidence of infections caused by this fungus is also very low. Most publications refer to isolated cases or series involving very few patients [9,10]. Although the incidence is very low, its high mortality rate in invasive infection (ranging from 40 to 80%) conditions causes its impact on solid organ recipients to be relevant [6,7].

*T. asahii* is the species most frequently implicated in severe invasive infections, although other species, such as *T. inkin* and *T. faecale*, can also be relevant pathogens [9,10,11]. The most common clinical manifestations include soft tissue infections, pneumonia, and fungemia. In some cases, there is involvement of the central nervous system and recurrent infections [3,5,7,12]. Infections caused by *Trichosporon* spp. present significant therapeutic challenges and are associated with poor clinical outcomes [6,7,13].

Intrinsic resistance to multiple antifungals complicates treatment [14]. *Trichosporon* spp. exhibit resistance to echinocandins, with variable susceptibility to amphotericin B and fluconazole. Voriconazole is the treatment of choice due to its superior in vitro activity and better-reported clinical outcomes [5,13,15]. However, accurate identification at the species level is essential, given differences in antifungal susceptibility and prognosis [3,5]. Removal of the central venous catheter is an essential therapeutic measure, given the tendency of *Trichosporon* spp. to form biofilms on medical devices [15].

We sought to describe a case series of SOT recipients with *Trichosporon* spp. infection and identify its prognostic factors. Understanding these factors is crucial to optimize the management of this infection and reduce its high mortality rate.

## 2. Materials and Methods

This is a retrospective analysis of invasive *Trichosporon* spp. infections in SOT recipients diagnosed between 2017 and 2025. The research was conducted at a tertiary hospital (Hospital Universitario Puerta de Hierro) with extensive experience in the management of SOT patients based in Majadahonda (Madrid) Spain. Cases were identified retrospectively by searching the Microbiology Laboratory database for all *Trichosporon* spp. isolates between January 2017 and September 2025. Subsequently, the electronic medical records of the corresponding patients were reviewed to collect demographic, clinical, treatment, and outcome data.

### 2.1. Microbiology

All clinical samples (sputum, blood, tissue, etc.) were collected following standard hospital protocols. They were immediately transported to the microbiology laboratory for processing. The samples were cultured on standard media such as Sabouraud dextrose agar and blood agar. Fungal isolates were stored at −80 °C in cryovials until further analysis. Microscopically, fungal structures compatible with *Trichosporon* spp. were observed, which were identified as *Trichosporon inkin* by Matrix Assisted Laser Desorption/Ionization Time-of-Flight Mass Spectrometry (MALDI-TOF MS) (Bruker Daltonik, Bremen, Germany). For better identification, isolates were sent to the Spanish National Center of Microbiology, and the identification was made by sequencing the ITS and IGS1regions from ribosomal DNA [16,17].

Antifungal susceptibility testing was performed using the method for determining broth dilution MICs of antifungal agents for fermentative yeast. EUCAST criteria were used [18]. Blood cultures were processed using the BACTEC™ automated system (Becton Dickinson, Franklin Lakes, NJ, USA). Viral loads were determined using the Quant CMV LightCycler 2.0 real-time PCR System (Roche Applied Science, Basel, Switzerland).

### 2.2. Variables

The variables studied were patient demographics (age, sex), type of solid organ transplant, retransplantation, comorbidities, characteristics of the infection (initial site, presence of fungemia, time to diagnosis, breakthrough status, and concomitant CMV replication > 500 copies/mL), clinical status at diagnosis (ICU admission, acute kidney injury, use of invasive devices, organ dysfunction, and multi-organ failure), laboratory values (neutrophil and platelet counts, tacrolimus levels), therapeutic management (immunosuppressive regimens, concomitant antibiotics, specific antifungal therapy, surgical debridement), and microbiological data, including *Trichosporon* species identification and in vitro antifungal susceptibility testing (MICs) for a panel of azoles, polyenes, and echinocandins.

### 2.3. Definitions

Chronic renal failure was defined as a glomerular filtration rate (GFR) of less than 60 mL/min/1.73 m^2^ maintained for more than three months. Acute renal failure was considered to have occurred if there was an increase in serum creatinine of ≥50% from baseline over the last 7 days. Surgical site infections were defined according to the criteria of the Centers for Disease Control and Prevention and the National Healthcare Safety Network. These were classified as superficial infections (affecting the skin and subcutaneous tissue), deep-incisional infections (affecting the fascia and muscle), and organ/space infections (affecting any part of the body manipulated during surgery, excluding the skin, fascia, and muscle) [11]. Fungemia was defined as positive blood cultures in one or more bottles. Breakthrough infection was defined as infection detected after at least five days of systemic treatment with an antifungal agent [19]. In-hospital infection-related mortality was defined as death during hospitalization due to *Trichosporon* infection.

### 2.4. Statistical Analysis

Demographic, clinical, and therapeutic data, as well as hospital mortality of the included patients, were analyzed. Qualitative variables are expressed as absolute numbers and percentages. Quantitative variables are expressed as median and interquartile range (IQR). Categorical variables were compared using Fisher’s test. Quantitative variables were compared using Mann–Whitney’s U. A two-sided *p*-value below 0.05 was considered significant.

## 3. Results

**Figure 1:**
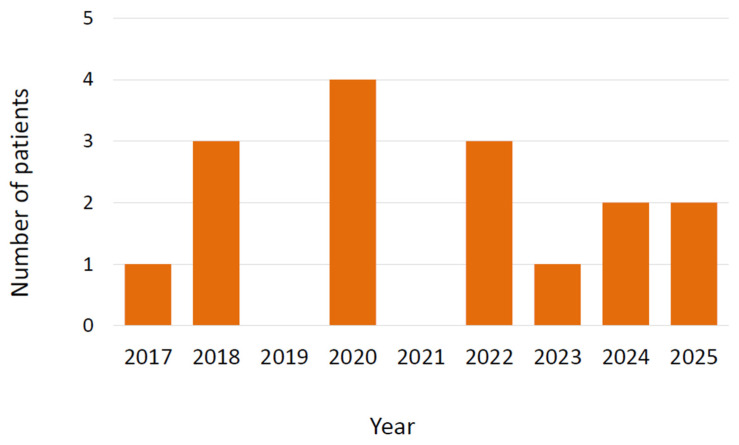
Cases of infection by *Trichosporon austroamericanum* in transplant recipients according to year of diagnosis.

During the study period, 16 cases of infection due to *Trichosporon* spp. were detected. The strains were identified as *T. inkin* using MALDI-TOF, but in the reference laboratory they were identified as *T. austroamericanum* by sequencing of the ITS and IGS regions of the ribosomal DNA due to its homology to the type strain of this species (CBS 17435). Nine patients were male (56.2%) and the median age was 54 years with an interquartile range (IQR) of 46.5 to 60 years. The type of organ transplanted was lung in 8 patients (50%, one unilateral lung transplantation 6.2%), heart in 5 patients (31.2%), and liver in 3 patients (18.7%). The proportion by type of transplant was 1.77% in lung transplant recipients (8 out of 452), 2.79% in heart transplant recipients (5 out of 179), and 1.14% in liver transplant recipients (3 out of 263). In 3 cases (18.7%), the patients had received a retransplant (2 lung, 1 liver). No cases were detected among the 10 patients who underwent cardiopulmonary transplantation or among the 250 kidney transplants. Figure 1 shows the chronological distribution of cases according to the year of diagnosis.

The median daily equivalent dose of prednisone was higher in patients who ultimately died (37.5 mg) than in survivors (18 mg, *p* = 0.020). Likewise, the administration of mycophenolate as a third immunosuppressive agent was observed in 100% of those who died and only in 50% of survivors (Table 1, *p* = 0.077).

In 14 cases (87.5%), the infection was initially located at the surgical site. In 6 patients (37.5%), the initial culture was from the surgical wound and in the other 8 patients (50%), it was isolated in the pleural fluid (lung transplantation, 5 patients; heart transplantation, 2 patients) or in ascites fluid (1 patient with liver transplantation) (Table 1). In 6 patients with surgical infection (42.9%), the infection spread to the bloodstream, resulting in the death of all of them. In the remaining 2 patients (12.5%), the first sign of infection was fungemia without evidence of the source of infection. The patients were not in the same ward at the same time, neither in the general ward nor in the ICU.

In all cases of surgical wound infection, surgical debridement was performed alongside antifungal treatment. The median time to presentation (between surgery and diagnosis of infection) was 35 days (IQR: 14.5–110.5 days). Notably, the time to presentation was significantly shorter in those who died, with a median of 16 days (IQR: 5.5–32 days), compared to 91 days (IQR: 15–183 days) in survivors (*p* = 0.005).

Of the 8 patients who developed primary or secondary fungemia, the catheter was removed and cultured in 5 of them, and only 1 case yielded *T. austromericanum*. Previous colonization by this yeast was detected in only 2 cases (14.3%). CMV replication was demonstrated in three non-survivors (42.9%) and two survivors (25%), a difference that was not statistically significant (*p* = 0.604). No other opportunistic infections were observed in these patients.

Azoles such as voriconazole, posaconazole, isavuconazole, and itraconazole have low MIC values (0.016–0.5 µg/mL), while fluconazole has higher MIC values (0.25–>8 µg/mL). Amphotericin B concentrates most strains in low and intermediate values (0.03–2 µg/mL), and echinocandins (caspofungin, anidulafungin, and micafungin) show no activity, with MIC > 8 µg/mL in 100% of strains.

Hospital mortality was 50% (8 patients). In addition to fungemia, other variables associated with hospital mortality in the univariate analysis that were present at the time of diagnosis included ICU stay, urinary catheter, central catheter, higher doses of glucocorticoids, and antibiotic treatment. Breakthrough infection was also associated with very high mortality (Table 1). The antifungal drugs these patients were taking prior to infection with *Trichosporon* were echinocandins in those who died and fluconazole in the patient who survived.

## 4. Discussion

Invasive infections by *Trichosporon* spp. in SOT recipients are a rare complication associated with high mortality. In our series, the infection was most commonly located at surgical site, but its progression to the bloodstream was associated with a very poor prognosis. Intensive care unit stay at diagnosis, high doses of corticosteroids, breakthrough infections, and concomitant antibiotic treatment were also associated with in-hospital mortality.

*Trichosporon* spp. are basidiomycete yeasts characterized by their ability to form robust biofilms on tissues and medical devices [8]. The isolates corresponded to a new emerging species of basidiomycete yeast named *T. austroamericanum*, molecularly identified using the intergenic spacer (IGS1) ribosomal DNA locus that was close to that of *T. inkin* [20]. Molecular analysis of the IGS1 locus is based on the amplification and sequencing of a highly variable region of ribosomal DNA, which allows closely related species to be differentiated [17]. On the other hand, MALDI-TOF MS identifies yeasts by analyzing protein profiles generated by mass spectrometry, comparing the spectra obtained with reference databases. Sequencing of the IGS region of the rDNA is the method of choice for the definitive identification and discrimination of cryptic species, although it requires more time and is only available in reference centers [17].

Although antifungal cut-off points have not been firmly established for *Trichosporon* spp., voriconazole is considered the treatment of choice, although other azoles such as isavuconazole or posaconazole are also considered appropriate [21]. In our series, we found that all strains were resistant to echinocandins, as has been repeatedly evidenced in previous publications [22,23].

*Trichosporon* infections in SOT recipients are extremely uncommon in Spain; to date, only one small series involving lung transplant recipients and a single case of a surgical infection has been documented [4,8]. They also remain a very rare infection in the rest of the world in SOT with most reports consisting of individual cases or reduced series [9,10]. We also do not consider transmission of the infection between patients possible, as they did not have close proximity during their hospital stay.

We should note that the strains from our patients had relatively low MICs to amphotericin B, which could give it a potential therapeutic role [11,15,21]. However, monotherapy with this antifungal is not recommended for infections due to possible discrepancies between in vitro sensitivity and doubts about its clinical efficacy. This has been associated with the formation of resistant biofilms, as well as with decreased affinity and membrane penetration of polyene antifungals, which could reduce their in vivo effect [6,13,24,25,26].

The high mortality rate of this infection (50% in our series) has been previously reported [4,7,10,27]. Most fungemia cases in our series developed as a consequence of a surgical infection. This finding indicates a pronounced vascular tropism, which likely contributes to the notably high mortality rate observed [28,29]. This same finding has been observed in another recent publication [4]. The poor prognosis of this infection has also been linked to *Trichosporon*’s ability to form biofilms on devices such as catheters [30]. Although infection of these devices has not been demonstrated in most of our patients with fungemia, other authors have observed that early catheter removal is associated with a very significant decrease in mortality in cases of bloodstream infection [15]. In our study, given the limited sample size, a multivariate analysis could not be performed; however, there is likely a close association between mortality, the short time to presentation, diagnosis in the ICU, and the presence of invasive devices.

Breakthrough infection was significantly associated with mortality. *Trichosporon* spp. has been characterized by its ability to cause this type of infection during echinocandin treatment [31,32]. The worse prognosis of breakthrough IFIs has been linked to the selection of more resistant fungi and late diagnosis [33,34]. They also tend to occur in patients with a higher degree of immunosuppression and who have invasive devices such as central catheters and urinary catheters, which are more common in patients admitted to the ICU [7,31,33,35,36,37]. Antibiotics may increase mortality by promoting excessive growth of this yeast in infected or colonized organs [5,7]. For all these reasons, it is very important to promote the judicious use of antibiotics and antifungals in patients treated in the ICU [38,39].

Deep immunosuppression may also have contributed to an unfavorable outcome [23,28]. In fact, the equivalent dose of prednisone at diagnosis was 37.5 mg in patients who ultimately died and 18 mg in survivors (*p* < 0.05). A marked proliferation and spread of *Trichosporon* spp. could be the result of high doses of corticosteroids [7,31,32,40]. In addition, triple immunosuppressive therapy with corticosteroids, calcineurin inhibitors, and mycophenolate showed a tendency toward a worse prognosis compared to dual therapy without mycophenolate. Minimizing the dosage of corticosteroids and the number of concurrent immunosuppressants may represent a modifiable prognostic factor.

With respect to early diagnosis, it is important to note that the role of biological markers in this condition has not been thoroughly investigated; further studies are needed to establish their clinical utility. There is no validated serological test based on the detection of antibodies in blood that is available for the clinical diagnosis of *Trichosporon* spp. infection. Monoclonal antibodies for the identification of *T. asahii* and *T. asteroides* antigens have been described, but these have been used mainly in experimental settings [41]. Although there are reported cases in which beta-D-glucan in the blood was elevated in patients with disseminated *Trichosporon* infection, other studies have observed lower sensitivity than other markers, such as cryptococcal antigen [42,43]. Blood polymerase chain reaction could play a role in the early diagnosis and monitoring of this infection, although there are no solid studies on this subject [44]. One line of research to be developed is the search for serum markers that could facilitate the early diagnosis and treatment of this infection [45]. There is no validated serological test based on the detection of antibodies in blood that is available for the clinical diagnosis of *Trichosporon* spp. infection. Monoclonal antibodies for the identification of *Trichosporon asahii* and *T. asteroides* antigens have been developed in the literature, but these have been used mainly in experimental laboratory assays and not as commercial serological tests for the detection of antibodies in patients.

## 5. Limitations

Some important limitations of this study are its retrospective design and the reduced number of patients included, which prevented a multivariate analysis of the independent association of certain variables with mortality. Furthermore, as this is a single-center study, the results obtained may not be applicable to institutions with different characteristics. Regarding treatment, due to the relatively low sample size, it has not been possible to analyze whether combined treatment with azoles and amphotericin B could offer a prognostic benefit over azole monotherapy. This is a question that remains to be addressed in future studies, including those that allow validated clinical breakpoints to be established for antifungal susceptibility. Despite the limited sample size, this series represents the largest number of transplanted patients reported to date.

## 6. Conclusions

Invasive *Trichosporon* infection in SOT recipients is associated with high mortality. Administration of higher-dose corticosteroids, as well as the presence of antibiotic and antifungal treatment at diagnosis, are associated with higher mortality. Early diagnosis and surgical treatment of localized infections could reduce mortality. The combined administration of broad-spectrum antibiotics and echinocandins was associated with mortality in transplant patients with *Trichosporon* spp. infection.

## Figures and Tables

**Table 1:** Characteristics of solid organ transplant recipients with *Trichosporon inkin* infection according to hospital mortality.

	Death (*n* = 8)	Survival (*n* = 8)	*p*
Age (years)	54.5 (47–59)	56 (49.5–67)	0.641
Male gender	4 (50)	5 (62.5)	1
Type of transplant			
Lung	5 (62.5)	3 (37.5)	0.619
Heart	2 (25)	3 (37.5)	1
Liver	1 (12.5)	2 (25)	1
Retransplantation	3 (37.5)	0	0.200
Diabetes mellitus	1 (12.5)	3 (37.5)	0.569
Chronic kidney failure	0	2 (25)	0.467
Acute kidney failure ^1^	7 (87.5)	3 (37.5)	0.118
HIV infection	0	1 (12.5)	1
Neutrophils/mm^3^, median (IQR)	8100 (5600–9696)	5830 (2500–8645)	0.440
Platelets 10^3^/mm^3^, median (IQR)	119.5 (66–183.5)	219 (134–314)	0.140
Initial site of infection			
Bloodstream	1 (12.5)	1 (12.5)	1
Surgical wound	2 (25)	4 (50)	0.608
Pleural fluid ^2^	4 (50)	3 (37.5)	1
Ascitic fluid ^2^	1 (12.5)	0	1
Positive blood cultures ^3^	7 (87.5)	1 (12.5)	0.010
Immunosuppression			
Corticosteroids	8 (100)	8 (100)	1
Equivalent dose (mg/day), median (IQR)	37.5 (30–80)	18 (11–27)	0.020
Tacrolimus	8 (100)	8 (100)	1
Plasma level of tacrolimus (mg/mL), median (IQR)	10.5 (6.8–16.1)	10.1 (7.7–13.9)	0.784
Mycophenolate	8 (100)	4 (50)	0.077
Transplanted organ disfunction	8 (100)	4 (50)	0.077
Transplanted organ rejection	2 (25)	1 (12.5)	1
ICU admission	8 (100)	1 (12.5)	0.001
Central catheter	8 (100)	3 (37.5)	0.026
Bladder catheterization	8 (100)	3 (37.5)	0.026
Breakthrough infection ^4^	7 (87.5)	1 (12.5)	0.010
Concomitant antibiotic	8 (100)	3 (37.5)	0.026
Antifungal treatment			
Voriconazole	1 (12.5)	3 (37.5)	0.569
Other antifungal drug	7 (87.5)	5 (62.5)	0.569
Isavuconazole	4 (50)	5 (62.5)	1
Posaconazole	2 (25)	0	0.467
Azole and amphotericin B	2 (25)	2 (25)	1
Time elapsed to antifungal treatment (days) ^5^, median (IQR)	4 (2–4.5)	2 (1.5–5)	0.641
Multi-organ failure	8 (100)	2 (25)	0.007

^1^ Acute kidney failure requiring renal replacement therapy during ICU stay. ^2^ All cultures in pleural or ascites fluids were related to surgical infection of an organ or space. ^3^ Positive blood cultures in any patient, including patients whose infection initially presented as a surgical site infection. ^4^ The antifungal the patients were receiving was an echinocandin in the deceased patients and fluconazole in the surviving patient. ^5^ Time elapsed from obtaining the culture to administering the appropriate antifungal agent. The analytical results correspond to the time of diagnosis of the infection.

## Data Availability

The original clinical data presented in the study and any other queries can be supplied by contacting the corresponding author. The original DNA sequencing data presented in the study are openly available at GenBank from NCBI with the following accession numbers: PX611853-PX611867 (ITS sequences) and PX644868-PX644882 (IGS sequences).
